# Distinct physical condition and social behavior phenotypes of CD157 and CD38 knockout mice during aging

**DOI:** 10.1371/journal.pone.0244022

**Published:** 2020-12-16

**Authors:** Maria Gerasimenko, Olga Lopatina, Anna A. Shabalova, Stanislav M. Cherepanov, Alla B. Salmina, Shigeru Yokoyama, Hisanori Goto, Hiroshi Okamoto, Yasuhiko Yamamoto, Katsuhiko Ishihara, Haruhiro Higashida

**Affiliations:** 1 Department of Basic Research on Social Recognition and Memory, Research Center for Child Mental Development, Kanazawa University, Kanazawa, Japan; 2 Laboratory for Social Brain Studies, Research Institute of Molecular Medicine and Pathobiochemistry, Krasnoyarsk State Medical University named after Prof. V. F. Voino-Yasenetsky, Krasnoyarsk, Russia; 3 Department of Biochemistry, Krasnoyarsk State Medical University named after Prof. V. F. Voino-Yasenetsky, Krasnoyarsk, Russia; 4 Department of Biochemistry and Molecular Vascular Biology, Kanazawa University Graduate School of Medical Sciences, Kanazawa, Japan; 5 Department of Immunology and Molecular Genetics, Kawasaki Medical School, Kurashiki, Okayama, Japan; Tokyo Metropolitan Institute of Medical Science, JAPAN

## Abstract

The ability of CD38 and CD157 to utilize nicotinamide adenine dinucleotide (NAD) has received much attention because the aging-induced elevation of CD38 expression plays a role in the senescence-related decline in NAD levels. Therefore, it is of interest to examine and compare the effects of age-associated changes on the general health and brain function impairment of Cd157 and Cd38 knockout (CD157 KO and CD38 KO) mice. The body weight and behaviors were measured in 8-week-old (young adult) or 12-month-old (middle-aged) male mice of both KO strains. The locomotor activity, anxiety-like behavior, and social behavior of the mice were measured in the open field and three-chamber tests. The middle-aged CD157 KO male mice gained more body weight than young adult KO mice, while little or no body weight gain was observed in the middle-aged CD38 KO mice. Middle-aged CD157 KO mice displayed increased anxiety-like behavior and decreased sociability and interaction compared with young adult KO mice. Middle-aged CD38 KO mice showed less anxiety and hyperactivity than CD157 KO mice, similar to young adult CD38 KO mice. The results reveal marked age-dependent changes in male CD157 KO mice but not in male CD38 KO mice. We discuss the distinct differences in aging effects from the perspective of inhibition of NAD metabolism in CD157 and CD38 KO mice, which may contribute to differential behavioral changes during aging.

## Introduction

Aging is associated with impairments in a wide range of physical and brain functions in humans and animals [1–7]. Furthermore, 10–15 month-old mice can be considered as middle-aged, during which time senescence processes and reproductive decline begin to occur [8, 9]. With aging, gains in body mass [10–13] and reduced locomotor activity are frequently observed [10, 12–16] (but see [17]). Additionally, some studies have reported increases in anxiety-related [12–14, 16] and depression-like behavior [16], as well as decreased social interaction [1, 12, 16]. During middle age, mice may begin to display a decline in cognitive function and memory loss [13, 15, 16, 18–20]. Although this does not always occur, especially in early middle-aged mice [12, 13], it is worth examining age-related changes in middle-aged mice instead of examining older mice (24 months).

Studies on autistic individuals mostly focus on children and/or young adult individuals and the same is true for model rodents. However, there is one article describing aging in BTBR mice–a mouse model of idiopathic autism spectrum disorder (ASD) examined at the age of 15 months, which is assigned as the highest border of middle-age [21]. They found decreased sociability in aged BTBR mice compared to aged wild-type (WT) mice. Nevertheless, locomotion activity and cognitive ability were comparable between genotypes. On the other hand, although aging is also less studied in ASD patients, it has been reported that ASD conditions are associated with premature death [22, 23], probably because of high incidence of the coexistence of physical diseases in such subjects [23]. Interestingly, it was found social cognition remains unchanged or improved in aging ASD individuals [24, 25]. Moreover, the prevalence of neuropsychiatric symptoms in older ASD subjects is likely to be lower relative to that of young adults [26, 27]. Nevertheless, data on aging-related cognitive decline is controversial, as fewer age-related cognitive changes are reported in ASD individuals [27–29], than neurotypical individuals [30].

CD38 and CD157 are immune-related molecules that share several enzymatic functions [31–34]. Both molecules possess cyclase activity and can metabolize nicotinamide adenine dinucleotide (NAD) into ADP-ribose and cyclic ADP-ribose (cADPR), which are important secondary messengers that can potentially activate transient receptor potential cation channel, subfamily M, member 2 (TRPM2) channels and trigger Ca^2+^ mobilization in the ryanodine receptor-sensitive Ca^2+^ pools [31–33, 35]. This increase in the intracellular free Ca^2+^ concentration has been shown to facilitate oxytocin release into the brain and the systemic circulation at the posterior pituitary gland [36, 37]. When cADPR-sensitive CD38-dependent oxytocin release is disrupted in mice with a null mutation in the Cd38 gene (CD38 KO mice), abnormal social behavior can be induced. CD38 KO pups on postnatal day 7 exhibited a higher level of locomotor activity and a lower number of ultrasonic vocalizations than WT pups [31, 38, 39]. Young adult (approximately 8–12 weeks old) CD38 KO mice showed increased locomotor activity, deficits in social memory reductions in anxiety-like behavior, and impairment in parental behavior [36, 40]. However, relatively little is known about the behavioral changes in aged CD38 KO mice.

In contrast, a Cd157 knockout (CD157 KO) mice, which is a model of pre-motor symptoms of Parkinson’s disease, can be characterized by presenting increased anxiety-related and depression-like behavior or social avoidance in young adulthood [41–44]. A decreased number of calls and a poor vocal repertoire were also detected in CD157 KO pups [45]. Additionally, little or no data exist regarding behavioral changes in aged CD157 KO mice.

Recently, the ability of CD38 to metabolize NAD has received much attention because elevated CD38 expression plays a role in the senescence-related decline in NAD levels [36–38]. Interestingly, aged CD38 KO mice have been reported to exhibit better general health than WT mice of the same age [46]. Moreover, inhibition of the catalytic activity of CD38 by small molecule inhibitors or monoclonal antibodies might also promote a beneficial effect by sustaining higher NAD levels in aged mice [47].

General health outcomes in aging CD157 KO mice have not been previously reported. Therefore, we first examined middle-aged CD157 KO mice and then determined similarities and differences in aging-induced changes between CD38 KO and CD157 KO mice, especially with respect to body weight and social behavior. CD38 KO and CD157 KO mice were studied simultaneously because their genotypes differ (Cd38^-/-^/Cd157^+/+^ and Cd38^+/+^/Cd157^-/-^, respectively), and we expected distinct outcomes because of the differential deletion of these NAD-related genes. The effects of these molecules on aging can be determined by directly comparing CD157 and CD38 KO mice without using aged WT mice as controls.

## Materials and methods

### Animals

Cd157/Bst1^-/-^ (CD157 KO on a C57BL/6N background) mice were described previously [40, 48]. CD157 KO mice were maintained by crossbreeding homozygous mutant mice. Cd38^−/−^ (CD38 KO) mice with an ICR genetic background were described previously [36, 49]. Most experiments were performed using a congenic method on selected adult males of the homozygous KO groups. All CD157 KO and CD38 KO mice were born in our laboratory colony. Pups were weaned at 21–28 days of age and housed in same-sex groups of five animals/cage in the animal center of our university under standard conditions (24°C; 12/12-h light/dark cycle, with lights on at 8:45 a.m.) with ad libitum access to food and water. The mice were fed with Charles River formula chow with a standard caloric content (Oriental Yeast Co. Ltd, Cat # CRF-1, Tokyo, Japan). Behavioral testing was performed on mice aged 8 or 12 weeks (n = 7–10 for CD157KO, and n = 5–10 for CD38KO) (designated as young adult) or 12 months (n = 6 for CD157KO and n = 4 for CD38KO) (designated as middle-aged). The body weight of each mouse was measured on a standard laboratory scale. Body mass index (BMI) was calculated by dividing the mouse’s body weight (grams) by the square of its length in centimeters from the nose tip to the anus [50]. All animal experiments were carried out in accordance with the Fundamental Guidelines for Proper Conduct of Animal Experiments and Related Activities in Academic Research Institutions under the jurisdiction of the Ministry of Education, Culture, Sports, Science and Technology of Japan and were approved by the Kanazawa University Committee on Animal Experimentation.

### Open field test

An Open field test was performed as described previously [44, 51]. Briefly, the open field chamber consisted of a square wooden box (550 × 600 × 400 mm), and the inner surfaces were covered with polypropylene sheets. The open field was divided into an inner zone (300 × 300 mm) and the periphery. First, a mouse was placed in the arena for 10 min (session 1, as a habituation period) and then returned to its home cage.

In session 2 (with a non-social object), an empty wire cage was placed in the center of the arena. The mouse was placed in the open field for 10 min, and during this time stress was induced by the non-social target. Then, the mouse was returned to its home cage.

In session 3, a naïve WT male mouse 8 weeks age (a C57BL6N mouse was used with CD157 KO mice, and an ICR mouse was used with CD38 KO mice) was placed in a wire cage at the center. The time spent in the inner and outer zones, total distance traveled, and immobility time were analyzed using a digital video system and ANY-maze video tracking software (Stoelting Co., Wood Dale, IL, USA). After each session, the test chambers were sprayed with 1% sodium hypochlorite and 70% ethanol and cleaned with paper towels [44]. The time interval between sessions was 2–3 min.

### Three-chamber test

A preference test for social targets of mice was performed using a three-chamber box [44]. The apparatus consisted of a rectangular, three-chambered box covered with clear polycarbonate. Dividing walls had doorways allowing access into each chamber. At the end of each test, the apparatus was sprayed with 1% sodium hypochlorite and 70% ethanol and wiped clean with paper towels. The following procedure was used for the social behavior test:

1. Habituation. The day before the test, mice were habituated in an empty apparatus for 20 min. Stranger mice were also habituated for 20 min in small cages. On the day of the experiment, the test mouse was first placed in the middle chamber and allowed to explore for 5 min with free access to all parts of the arena. Each of the two sides contained an empty wire cage (70 mm × 90 mm × 70 mm with bars spaced 5 mm apart). Zones located at 2.5 cm intervals around the wire cages were considered zones of direct interaction (cage zone)
2. Sociability. After habituation, an unfamiliar mouse (Stranger 1 [Str1] a naïve C57BL/6 male) was placed in the wire cage (in the left chamber). The other wire cage (in the right chamber) was left empty, and the test mouse was placed in the center compartment of the social test box and allowed to explore for a 5-min session, with free access to the two side chambers. The amount of time spent in the cage zone in each chamber was measured using a digital video system and ANY-maze software.
3. Social novelty preference. At the end of the 5-min sociability test, each mouse was further tested in the third 5-min session to quantitate preference for spending time with a new stranger. The new unfamiliar mouse (Str2; an experiment-naïve C57BL/6 male mouse) was placed in the wire cage (in the right chamber) that was empty during the previous 5-min session. The test mouse had a choice between the first, already-investigated, now-familiar mouse (Str1) and the novel unfamiliar mouse (Str2).

As described above, the amount of time spent in each chamber and in the direct interaction zones was measured using a digital video system and ANY-maze software. At the end of each test, the three-chamber box was cleaned as described above. The mean time interval between sessions was 2–3 min.

### Statistical analysis

GraphPad Prism 6 (GraphPad Software, San Diego, CA, USA) was used for statistical analysis. Data are expressed as the mean ± S.E.M. Comparisons were performed between young adult and middle-aged mice. The statistical analysis was performed by multiple Student’s t-tests or two-way analysis of variance (ANOVA) followed by post hoc Bonferroni tests. In all analyses, p < 0.05 indicated statistical significance.

## Results

**Fig 1:**
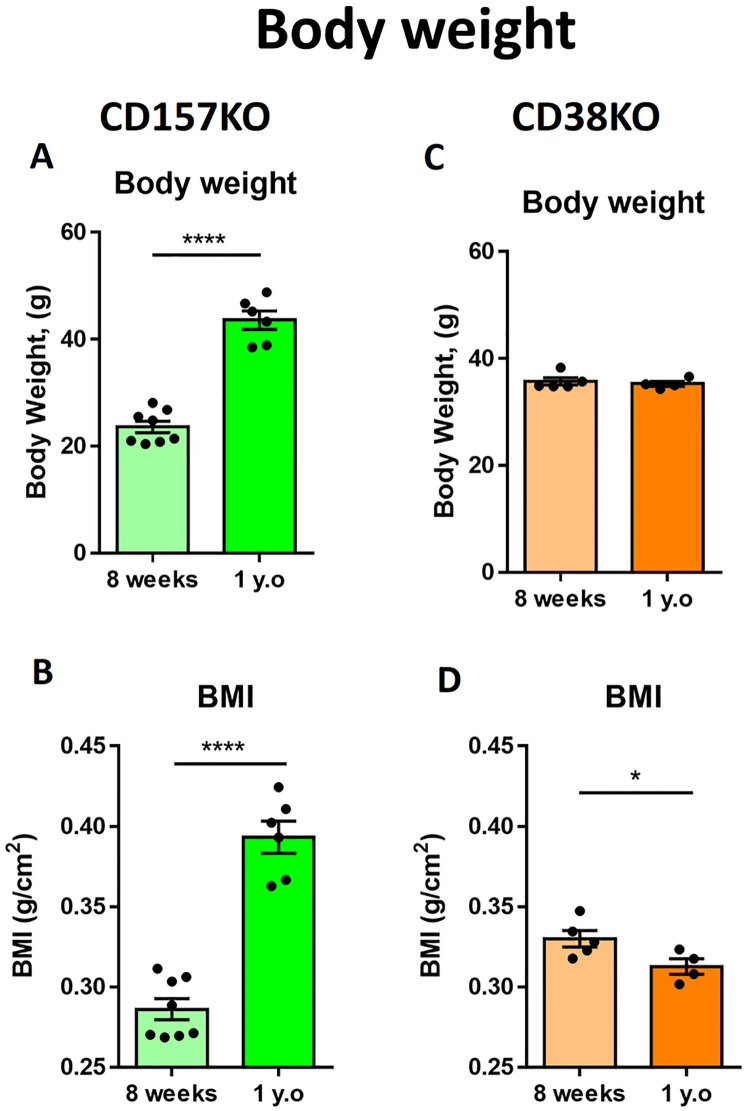
Body weight and body mass index (BMI) of young adult (8-week-old) and middle-aged (1-year-old) mice. (A) Body weight of CD157 KO mice. (B) BMI of CD157 KO mice. (n = 8 for young adult, n = 6 for middle-aged) (C) Body weight of CD38 KO mice. (D) BMI of CD38 KO mice (n = 5 for young adult, n = 4 for middle aged). Unpaired t-test, * p < 0.05, **** p < 0.0001.

We examined the age-dependent body weight gain in male CD157 and CD38 KO mice (Fig 1). Twelve-month-old (designated as middle-aged) CD157 KO mice had a significantly higher body weight (Fig 1A) and body mass index (BMI; Fig 1B) than 8-week-old mice (young adult): t_(12)_ = 10.37, p < 0.0001 for body weight; t_(12)_ = 9.369, p < 0.0001 for BMI.

In sharp contrast, middle-aged (12 month-old) CD38 KO male mice had a similar range of body weight to that of young adult mice (8 weeks old) (Fig 1C; t_(7)_ = 0.4878, p = 0.6406). The BMI of middle-aged CD38 KO mice was slightly but significantly lower than that of young adult mice (Fig 1D; t_(7)_ = 2.417 p = 0.0463). Therefore, middle-aged CD38 KO mice did not gain body mass but rather exhibited a decrease in body mass, while middle-aged CD157 KO mice gained weight with age.

### Locomotion and anxiety-related behavior in a new environment in the open field test

**Fig 2:**
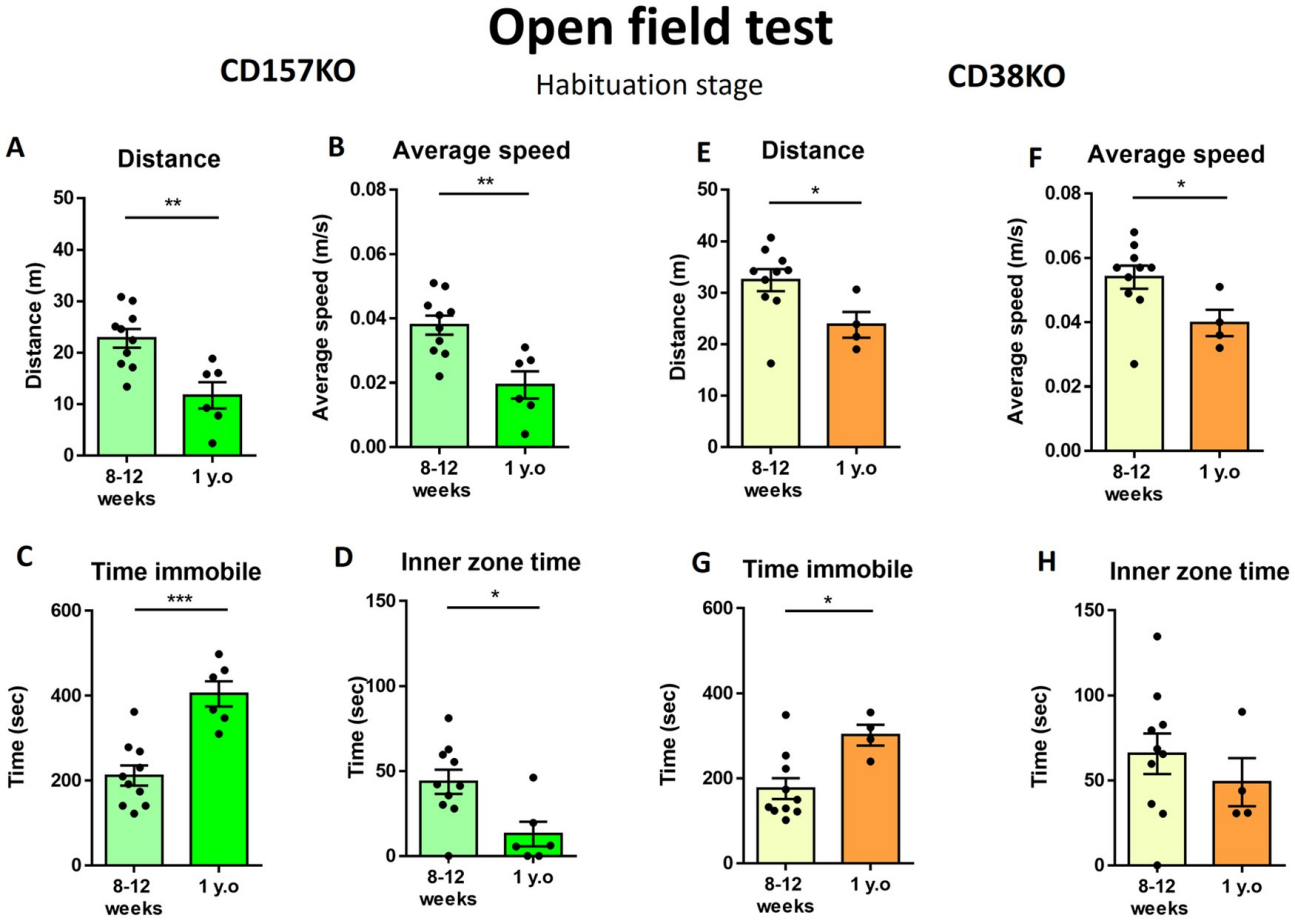
Locomotor activity in the open field test during the habituation stage. Values indicate the distance traveled in the arena (A), average speed (B), time spent immobile (C), and time spent in the inner zone (D) in young adult and middle-aged CD157 KO mice. (n = 10 for young adult, n = 6 for middle-aged). Values indicate the distance traveled in the arena (E), average speed (F), time spent immobile (G), and time spent in the inner zone (H) in young adult and middle-aged CD38 KO mice. (E) Distance of CD38 KO mice traveled in the arena. (F) Average speed of CD38 KO mice. (n = 10 for young adult, n = 4 for middle-aged) Unpaired t-test, *p < 0.05, **p < 0.01, ***p < 0.001.

Next, we measured the locomotor activity and social behavior in an open field test (S1 Fig). Anxiety-related behavior was examined in the habituation stage of the open field test, in which mice were exposed to a new environment. The distance traveled (Fig 2A) and average speed (Fig 2B) were significantly lower in middle-aged male CD157 KO mice than in young adult male CD157 KO mice (t_(14)_ = 3.637, p = 0.0027 for distance; t_(14)_ = 3.683, p = 0.0025 for average speed). The immobilization time was also longer in middle-aged mice (Fig 2C; t_(14)_ = 5.006, p = 0.0002). Middle-aged CD157 KO mice remained near the wall of the open field and are less likely to cross the central area. Thus, the average time in the inner zone was significantly lower in middle-aged mice (Fig 2D; t_(14)_ = 2.8370, p = 0.0132).

CD38 KO male mice displayed a similar pattern of locomotion with aging to CD157 KO mice, including less distance traveled in the arena (Fig 2E; t_(12)_ = 2.300, p = 0.0402), a lower average speed (Fig 2F; t_(12)_ = 2.260, p = 0.0432), and a longer immobility time (Fig 2G; t_(12)_ = 2.979, p = 0.0115). Middle-aged and young adult CD38 KO mice remained in the center area for a similar amount of time, as evidenced by the lack of a significant difference between the two groups (Fig 2H; t_(12)_ = 0.7930, p = 0.4432). The results showed that middle-aged CD157 KO mice displayed decreased locomotor activity and severe anxiety-related behavior, while middle-aged CD38 KO mice displayed reduced locomotion with less anxiety-related behavior.

### Anxiety-related behavior in the open field test with a non-social object

**Fig 3:**
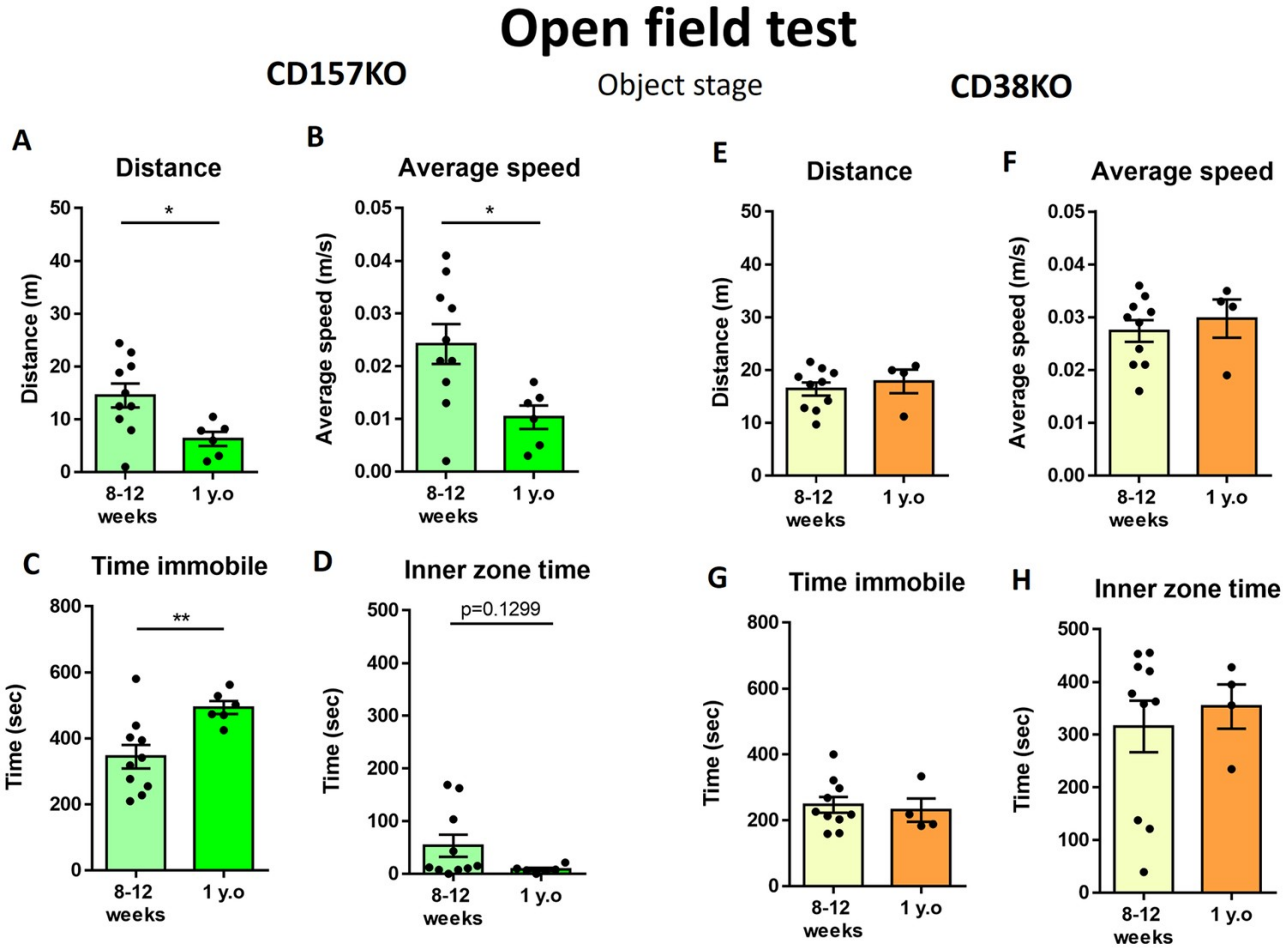
Behavior in the open filed test when a non-social object was placed in the center. Values indicate the distance traveled in the arena (A), average speed (B), time spent immobile (C), and time spent in the inner zone (D) in young adult and middle-aged CD157 KO mice. (n = 10 for young adult, n = 6 for middle-aged). Values indicate the distance traveled in the arena (E), average speed (F), time spent immobile (G), and time spent in the inner zone (H) in young adult and middle-aged CD38 KO mice. (n = 10 for young adult, n = 4 for middle-aged) Unpaired t-test, *p < 0.05, **p < 0.01.

When a non-social object (empty wire cage) was placed in the center area of the open field, the locomotor activity of middle-aged CD157 KO mice was similar to that observed in the habituation stage: distance traveled (Fig 3A; t_(14)_ = 2.611, p = 0.0205), average speed (Fig 3B; t_(14)_ = 2.652, p = 0.0189), and time spent immobile (Fig 3C; t_(14)_ = 3.027, p = 0.0091). Additionally, middle-aged CD157 KO mice tended to spend less time in the center zone of the arena (Fig 3D; t_(14)_ = 1.609, p = 0.1299), but not significantly.

In contrast, when a non-social object was placed in the center of the open field arena, middle-aged CD38 KO mice exhibited a locomotor activity level similar to that of young adult KO mice: distance traveled (Fig 3E; t_(12)_ = 0.6049, p = 0.5565), average speed (Fig 3F; t_(12)_ = 0.5904, p = 0.5659), time spent immobile (Fig 3G; t_(12)_ = 0.3589, p = 0.7259), and time spent in the inner zone (Fig 3H; t_(12)_ = 0.4551, p = 0.6571). Additionally, in the presence of the non-social object, age-dependent changes were observed in middle-aged CD157 KO mice, but such changes were not present in middle-aged CD38 KO mice.

### Anxiety-related behavior in the open field test with a social object

**Fig 4:**
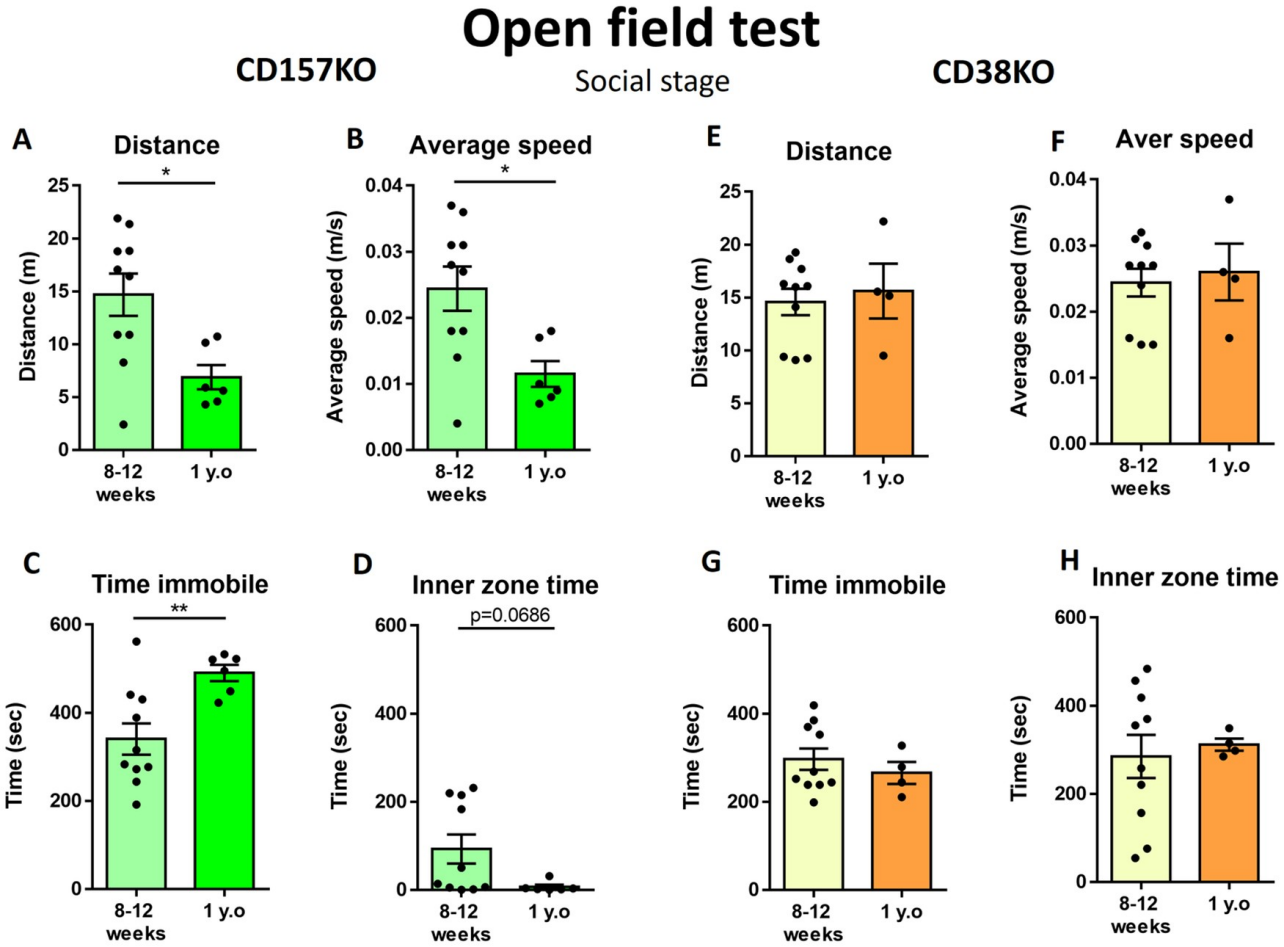
Behavior in the open field test when a social target was placed in the center. Values indicate the distance traveled in the arena (A), average speed (B), time spent immobile (C), and time spent in the inner zone (D) in young adult and middle-aged CD157 KO mice (n = 10 for young adult, n = 6 for middle-aged). Values indicate the distance traveled in the arena (F), average speed (F), time spent immobile (G), and time spent in the inner zone (H) in young adult and middle-aged CD38 KO mice. (n = 10 for young adult, n = 4 for middle-aged). Unpaired t-test, *p < 0.05, **p < 0.01.

Social behavior was assayed in the third stage of the open field test. The social object (an unfamiliar WT mouse of the same sex) was placed in a wire cage at the center of the arena (Fig 4). The middle-aged CD157 KO mice displayed a significantly decreased distance traveled (Fig 4A; t_(14)_ = 2.827, p = 0.0135), lower average speed (Fig 4B; t_(14)_ = 2.793, p = 0.0144), and increased time spent immobile (Fig 4C; t_(14)_ = 3.088, p = 0.0080). Moreover, the middle-aged CD157 KO mice remained in the inner zone of the arena for a markedly shorter time, although this was not statistically significant (Fig 4D; t_(14)_ = 1.973, p = 0.0686).

Surprisingly, the middle-aged male CD38 KO mice displayed nearly the same level of activity as young adult KO mice in the social stage of the open field test. The parameters in Fig 4E–4H were indistinguishable between young and middle-aged mice: distance traveled (Fig 4E; t_(12)_ = 0.4042, p = 0.6931), average speed (Fig 4F; t_(12)_ = 0.3755, p = 0.7138), time immobile (Fig 4G; t_(12)_ = 0.7427, p = 0.4720), and time spent in the inner zone (Fig 4H; t_(12)_ = 0.3348, p = 0.7436).

It was clear that in all stages of the open field test, the anxiety-related behavior was more pronounced in middle-aged CD157 KO male mice than in young adult CD157 KO mice. However, although middle-aged male CD38 KO mice displayed anxiety-related behavior during the habituation stage, no age-associated changes were observed in middle-aged CD38 KO mice in the open field test with the non-social and social targets.

### Sociability test in a three-chamber apparatus

**Fig 5:**
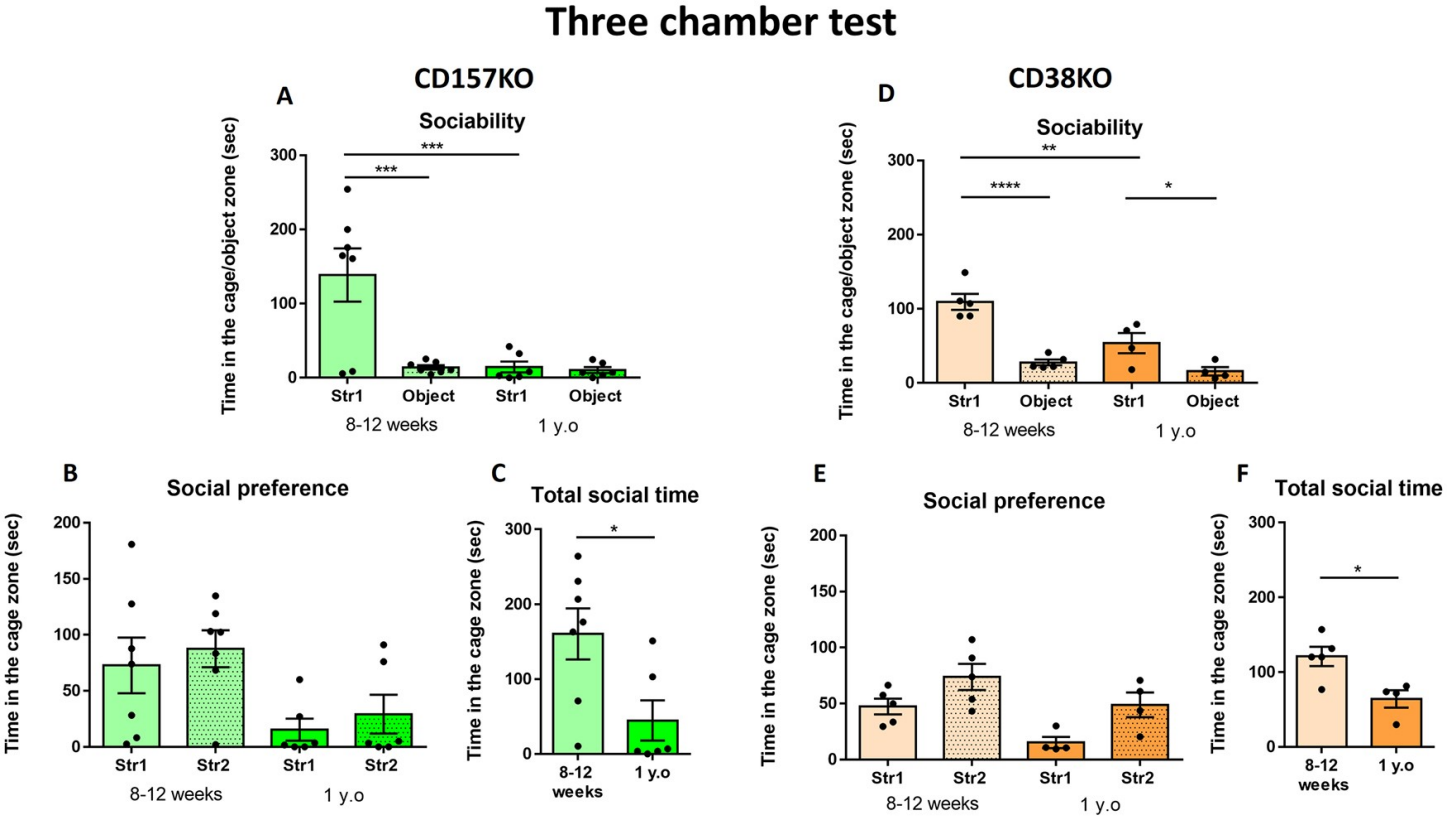
Social behavior in a three-chamber test in young adult and middle-aged CD157 KO mice (A–C) and CD38 KO mice (D–F). (A, D) The sociability stage. Time spent with a social (Str1) or a non-social object (Object). (B, E) The social novel preference stage. Time mice spent with a familiar mouse (Str1) or a novel mouse (Str2). (C, F) Total social time during which mice stayed in the arena with mouse targets. (In CD157KO mice, n = 7 for young adult, n = 6 for middle-aged, in CD38KO mice) Bonferroni’s post hoc comparison or Unpaired t-test, *p < 0.05, **p < 0.01, ****p < 0.0001.

A three-chamber test was performed to evaluate social behavior (Fig 5). In the sociability stage, mice usually choose to stay with a social target rather than a non-social target [52]. In CD157 KO mice (Fig 5A), two-way ANOVA showed a significant effect of age (F_(1,22)_ = 10.19, p = 0.0042), effect of object (F_(1,22)_ = 10.38, p = 0.0039) and age × object interaction (F_(1,22)_ = 9.097, p = 0.0064). Young adult male CD157 KO mice spent significantly more time with the mouse (Str1) than with the object (Bonferroni’s post hoc comparison p < 0.001). Middle-aged CD157 KO mice spent equal time with the social and non-social objects. Moreover, the time spent with Str1 by middle-aged CD157 KO mice was significantly shorter than that for young adult mice (Bonferroni’s post hoc test p < 0.001).

For CD38 KO mice (Fig 5D), two-way ANOVA revealed a significant effect of age (F_(1,14)_ = 13.65, p = 0.0024), effect of object (F_(1,14)_ = 42.77, p < 0.0001), and age × object interaction (F_(1,14)_ = 5.721, p = 0.0314). CD38 KO mice of both ages spent more time with the social object than with the non-social object (Bonferroni’s post hoc p < 0.0001 for young adult mice, p < 0.05 for middle-aged mice). The interaction time with Str1 by middle-aged CD38 KO mice was shorter than that for young adult KO mice (Bonferroni’s post hoc p < 0.01). The results showed that in male CD38 KO mice, the interest in both social and non-social targets seems to decrease in middle age, but sociability was not lost, even at middle age.

### Social novelty preference in the three-chamber test

Mice usually show interest in new social targets, which is termed a social novelty preference. Both young adult and middle-aged CD157 KO mice spent nearly equal time with Str1 and Str2, although the time spent in the cage with a new target mouse (Str2) was slightly longer than that spent with the familiar mouse (Str1), without being statistically significant (Fig 5B; two-way ANOVA revealed a significant effect of age (F_(1,22)_ = 9.647, p = 0.0052) but not an effect of object (F_(1,22)_ = 0.5934, p = 0.4493), or an object × age interaction (F_(1,22)_ = 0.0007, p = 0.9793). Regarding the total time spent in the social contact zones (total time mouse spent in contact with stranger mice), the time spent by middle-aged CD157 KO mice was significantly shorter than that of young adult mice (Fig 5C; t_(11)_ = 2.601, p = 0.0246), indicating that social interaction significantly decreased with aging.

In CD38 KO mice, two-way ANOVA showed a significant effect of age (F_(1,14)_ = 9.244, p = 0.0088) and object (F_(1,14)_ = 10.29, p = 0.0063), but not an age × object interaction (F_(1,14)_ = 0.1537 p = 0.7009). Both age groups of CD38 KO mice spent the same time with Str1 and Str2. Nevertheless, less total time was spent in the social contact zones by middle-aged mice (Fig 5F; t_(7)_ = 3.182, p = 0.0154).

The results of the three-chamber test indicate that middle-aged CD157 KO mice did not display sociability and social novelty preference; however, middle-aged CD38 KO mice remained sociable and showed a tendency of social novelty preference, similar to WT mice.

Additionally, aged CD157 KO mice displayed decreased locomotion activity in the sociability and social preference stages (S3A and S3B Fig). Locomotion activity of middle aged CD38KO was comparable to young adult (S3C and S3D Fig).

## Discussion

**Fig 6:**
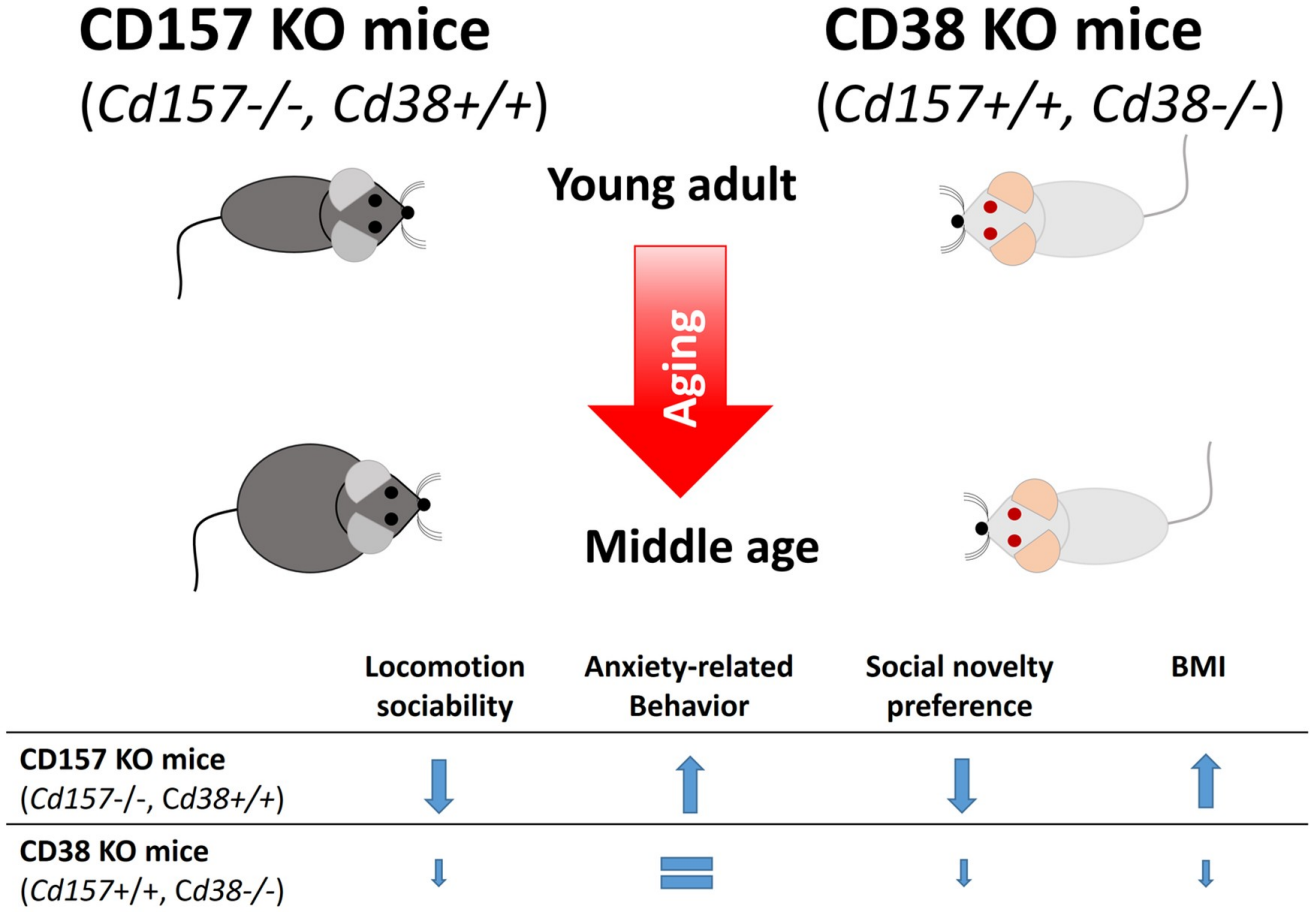
A scheme depicting changes observed in young adult and middle-aged CD157 KO and CD38 KO mice.

In the present study, we demonstrated the effect of aging on mice lacking CD157 or CD38 using a social behavioral test battery and BMI as a parameter of physical condition, as summarized in Fig 6. Because of differences in the genetic background (CD157 KO mice had a C57BL6 background and CD38 KO mice had an ICR background), it is difficult to perform a simple comparison between these two KO models, but changes induced by aging within the same strain can be observed.

Because no reports of middle-aged CD157 KO mice exist to our knowledge, it is not possible to compare our current results with other reports. Aging effects in CD38 KO mice on the other hand have been well studied [46, 53], but these mice were not produced on an ICR genetic background. Few reports are available regarding aging in the ICR strain [31, 36, 49]. In this respect, the current report is unique.

For animals, including laboratory mice, an increase in body weight associated with increasing fat mass is a relatively natural phenomenon during healthy aging [54, 55]. In our study, middle-aged male CD157 KO mice had higher body weight and BMI values than young adult mice. The weights of middle-aged (12 months old) C57BL6 male mice have previously been reported to be 34.4 ± 4.6 g [56] or 39.6 ± 4.7 g (The Jackson Laboratory https://www.jax.org/jax-mice-and-services/strain-data-sheet-pages/body-weight-chart-aged-C57BL6). Male middle-aged (1 year old) CD157 KO mice (on the C57BL6 genetic background) had a high body weight (43.6 ± 4.2 g). However, this value cannot be simply compared with reported weights of middle-aged WT C57BL6 mice because the environment and chow composition are not same. It is necessary to examine middle-aged CD157 KO mice and C57BL6 mice under the same conditions to investigate obesity in the future.

Body weight gain is an important issue. We performed a preliminary experiment to examine this issue by obtaining CD38 KO mice on a C57BL6 background from the Jackson Laboratory, designated as CD38BL6 KO, for two reasons. First, use of CD38BL6 KO mice on a C57BL6 background allows comparisons with CD157 KO mice. Additionally, in an independent experiment, mice were fed in a different location with similar normal chow. The mean body weights of 9-month-old mice were 34.6 ± 2.2 g in the C57BL6 group (n = 6), 38.0 ± 2.9 g in the CD157 KO group (n = 6), and 33.7 g in the CD38BL6 KO group (n = 6). C57BL6 and CD157 KO mice had similar body weights at the age of 8 weeks, but at the age of 9 months, the KO mice exhibited a greater body weight (S2 Fig; two-way ANOVA revealed a significant effect of age F_1,16_ = 100.5, p < 0.0001 but a marginal effect of genotype, F_1,16_ = 4.043, p = 0.0615 and genotype × age interaction, F_1,16_ = 2.571, p = 0.1284). CD157 KO mice tended to gain more body weight than initially expected.

Although middle-aged male CD38 KO mice had the same body weight as young adult KO mice, their BMI was significantly lower. According to recent studies, CD38 KO mice are resistant to a high-fat diet [57] or a high-fat, high-sucrose diet [58], both of which usually induce obesity in WT mice. It can be assumed that CD38 deficiency leads to increased NAD levels, which results in activation of NAD-dependent pathways that can suppress adipocyte differentiation and lipogenesis in adipose tissue [57].

The most important information obtained from the BMI is related to mouse adiposity, but the results are not always consistent. For example, in one study, the BMI was highly correlated with the amount of dissected adipose tissue [59], but in another study, such a correlation was not observed [60]. A lower BMI in middle-aged CD38 KO mice indicates lower adiposity; however, further examination of lipids in CD38 KO mouse tissues is required.

In ongoing experiments, CD157 KO mice are being fed with a high-fat, high-calorie diet rather than normal chow to determine whether CD157 KO mice show resistance to diet-induced obesity.

Middle-aged mice usually display less locomotor activity than young mice [10, 12–16]. However, middle-aged mice have been shown to have similar muscular strength to young mice [11, 12, 61]. Therefore, the decreased locomotor activity in KO mice may reflect an emotional abnormality rather than a physical weakness. This may be applicable to middle-aged CD157 KO mice, which showed decreased locomotor activity and increased time spent immobile, compared with young adult KO animals, in all stages of the open field test.

Recent studies have shown that young CD157 KO mice display deficits in social behavior [41, 44] (Fig 6). In the three-chamber test, young adult CD157 KO mice displayed typical rodent behavior in the sociability stage (choice between social and nonsocial behavior), but at one year of age, CD157 KO mice spent equal time with the social and nonsocial objects. In the social preference stage, young CD157 KO mice could not discriminate between a familiar and a novel social object, as previously reported [41]. The same pattern was observed for middle-aged CD157 KO mice, but this age group spent markedly less time near the social object. These results indicate social deficits or avoidance behaviors in CD157 KO mice.

Additionally, another study hypothesized that the onset of a social behavior decline in middle-aged mice may precede the cognitive deficits during aging [16]. However, further investigation is required, because we did not assess cognitive function in middle-aged mice in our study. Furthermore, a recent study showed that high adiposity in middle-aged mice may be accompanied by cognitive impairment [53]. In this regard, verification of the association of obesity status and cognitive function with a NAD-dependent metabolic disturbance could clarify this point in future, especially in middle-aged or older CD157 KO mice.

As shown in Fig 6, we could show clear differences between two KO mice in physical and behavioral changes associated with aging. Put simply, CD38 KO mice retained relatively younger phenotypes than CD157 KO mice at 12 months of age. Cellular CD38 expression is shown to increase with aging in WT mice, which results in a decrease in the cellular NAD content [46]. This condition is proposed to be a senescent state [46, 62]. Therefore, for anti-aging purposes, small molecule functional inhibitors of CD38 or anti-CD38 antibodies, which can neutralize CD38 function, are developed [63], as unlike CD38 KO mice, it may not be realistic to genetically nullify CD38 as a part of genetic therapy in humans. In CD157 KO mice, degradation of NAD by NAD glycohydrolase and ADP-ribosyl cyclase activities of CD157 is nullified, since CD38 is intact, and CD38 expression levels may increase with age. This may lead to the decreased NAD levels in middle-aged CD157 mice. These metabolic differences may cause in part behavioral and physical differences in the two mouse models, reflecting differences in NAD metabolism.

Our study comprises several important limitations. Firstly, CD157 KO and CD38 KO mice were based on different genetic backgrounds, C57BL6 and ICR, respectively. These mice strains possess considerable differences in behavior and physiology [64]. Although taking into account the universal effects of aging on rodent behavior, the strain difference can still significantly affect our results, resulting in difficulty in direct comparisons between CD157 KO and CD38 KO mice. Secondly, in this study we used only male mice. Absence of female results essentially limits our study. Future studies are required to clarify these points.

## Conclusion

In the current study, we revealed dramatic changes in social behavior in middle-aged CD157 KO mice and only a slight change in CD38 KO mice. In this case, the obvious marked decline in social behavior in middle-aged CD157 KO mice may indicate initial social behavior abnormalities, such as the ones observed recently in young adult mice [41] processes during the maturation/or the aging process. This process seems to be accelerated by increased CD38 expression, as observed in the cells during aging [65]. This hypothesis is supported by our results because relatively little change with aging was observed in middle-aged CD38 KO mice, likely because of the lack of CD38.

## Supporting information

S1 Fig Representative plots (typical example) of CD157KO and CD38KO mice in the open field test. (TIF)

S2 Fig Body weight of BL6 and CD157KO mice at the age of 8 weeks and 9 months. Bonferroni’s post hoc comparison, **p < 0.01, ****p < 0.0001.
(TIF)

S3 Fig Locomotion activity of CD157KO (A, B) and CD38KO (C, D) mice in the three chamber test. Unpaired t-test, *p < 0.05, **p < 0.01.
(TIF)
