# Supplementary figures and images for: Distinct physical condition and social behavior phenotypes of CD157 and CD38 knockout mice during aging
Source: PLoS One. 2020 Dec 16;15(12):e0244022. doi: 10.1371/journal.pone.0244022 (PMC7743928; doi:10.1371/journal.pone.0244022)

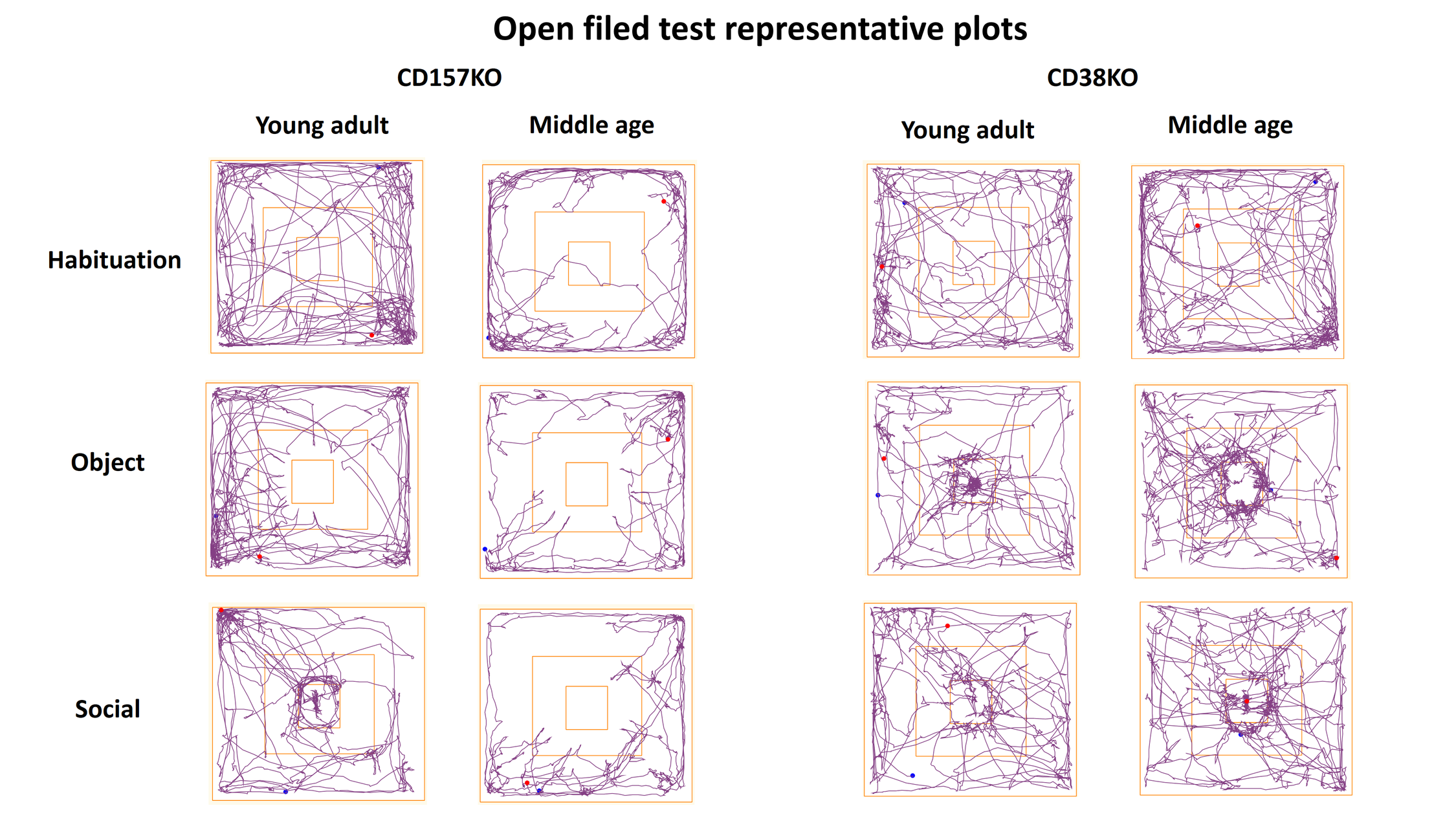

Supplement: S1 Fig — (TIF) [file pone.0244022.s001.tif]

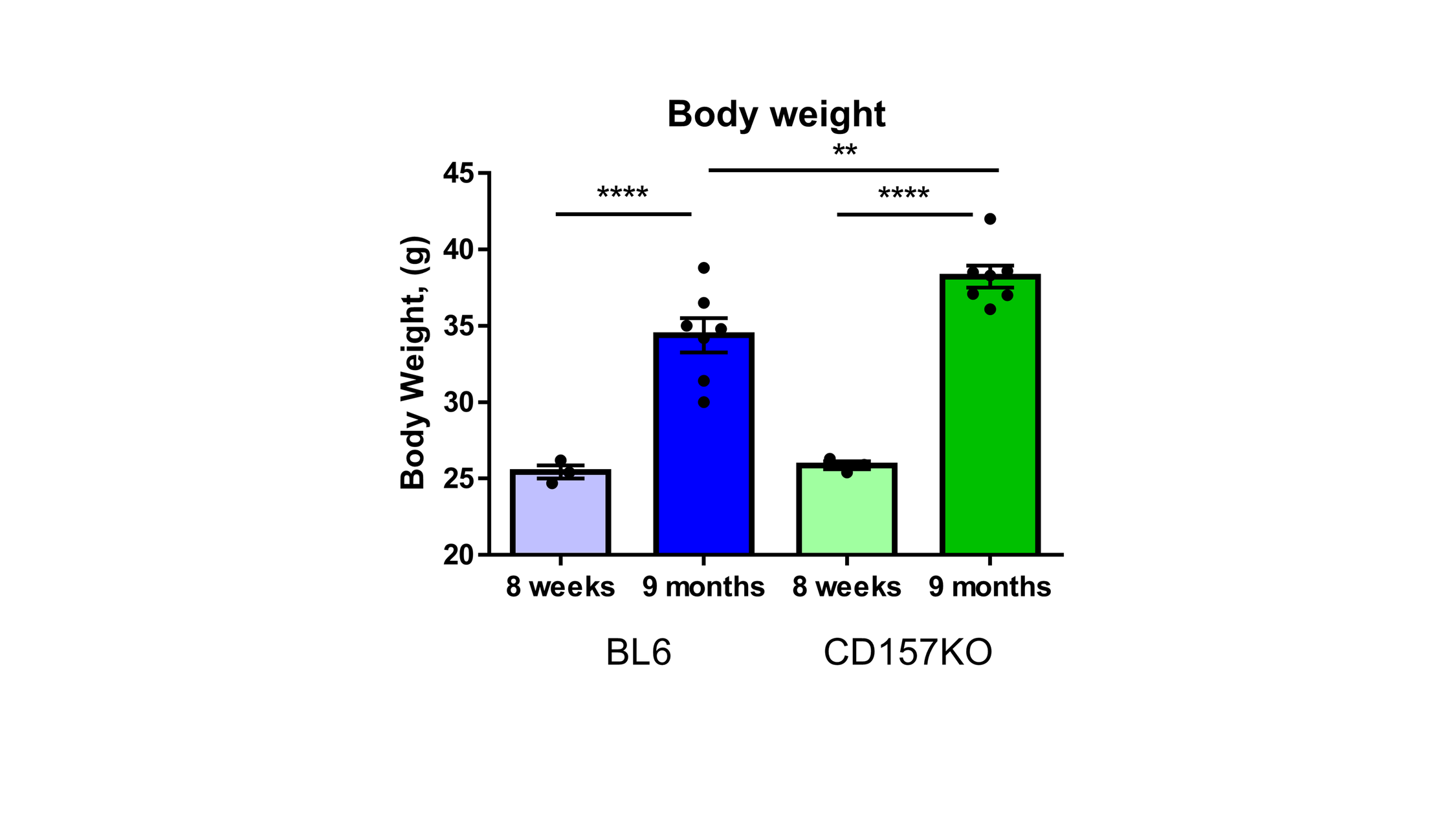

Supplement: S2 Fig — Bonferroni’s post hoc comparison, **p < 0.01, ****p < 0.0001. (TIF) [file pone.0244022.s002.tif]

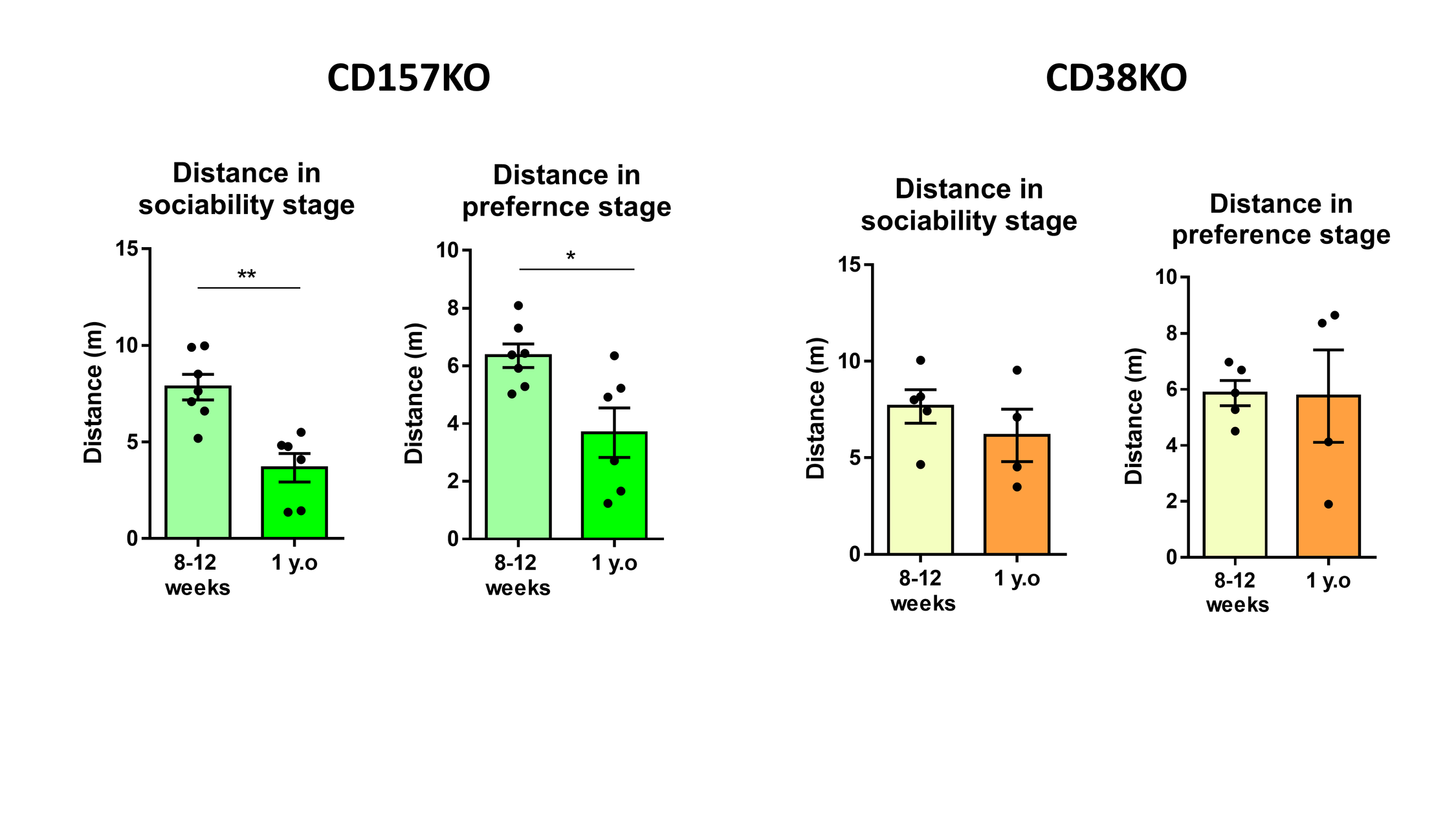

Supplement: S3 Fig — Unpaired t-test, *p < 0.05, **p < 0.01. (TIF) [file pone.0244022.s003.tif]
